# Meta-analysis on intervention effects of exercise on Chinese children and adolescents with mobile phone dependence

**DOI:** 10.3389/fpsyg.2025.1487944

**Published:** 2025-04-11

**Authors:** Long Chen, Yilin Ren, Pingting Zhu, Yahui Yang, Fengshu Zhu

**Affiliations:** ^1^College of Physical Education, Yangzhou University, Yangzhou, Jiangsu, China; ^2^College of Sports, Zhuhai Research Center for Women and Children’s Sports Culture, Jinan University Zhuhai Campus, Zhuhai, Guangdong, China; ^3^School of Nursing· School of Public Health, Yangzhou University, Yangzhou, Jiangsu, China

**Keywords:** exercise, mobile phone dependence, intervention effect, meta-analysis, exercise effect

## Abstract

**Objective:**

This study aims to examine which exercise regimens may have a stronger intervention effect and assess the impact of exercise on children’s and teenagers’ dependence on mobile phones using a meta-analysis system.

**Methods:**

From the library’s founding until December 2023, we searched the databases of China Knowledge, Wanfang, Wipro, PubMed, Web of Science, and the Cochrane Library for experimental studies on the effects of exercise on children’s and adolescents’ dependence on mobile phones. We also evaluated the literature’s quality using the Cochrane Handbook and performed meta-analyses using the RevMan 5.4 software. Using the standardized mean difference (SMD) and 95% confidence interval (CI) as the primary effect indicators, a meta-analysis was conducted utilizing RevMan 5.4 software. The protocol of this systematic review was registered in PROSPERO (CRD42024543710).

**Results:**

(1) Aerobic exercise showed a significant intervention effect on mobile phone dependence in children and adolescents in the intervention content subgroups but was not statistically significant in the combined exercise subgroups. (2) Exercise demonstrated a large effect size intervention effect on mobile phone dependence in children and adolescents. (3) Regarding mobile phone dependence in children and adolescents, the intensity of the intervention was divided into three subgroups: medium, moderate to high, and low. (4) The greatest intervention effect on mobile phone dependence was observed with a three-times-weekly intervention frequency. (5) Exercise lasting between 8 to 30 weeks showed a positive intervention effect on mobile phone dependence, with the most effective intervention occurring around 12 weeks. (6) The following order reflected the effect of the intervention duration on children and adolescents’ mobile phone dependence: 40–45 min, 20–30 min, ≥90 min, and 60 min.

**Conclusion:**

(1) Children and teenagers with cell phone addictions can benefit from exercise in reducing their symptoms; (2) the content, intensity, period, frequency and duration of exercise all exert a varying degree of influence on the intervention effect of exercise on mobile phone dependence in children and adolescents. Moderate-intensity aerobic exercise, lasting for 40–45 min three times a week for a total of approximately 12 weeks, is more likely to achieve the desired intervention effect of improving mobile phone dependence in children and adolescents.

## 1 Introduction

The obsessive condition of mobile phone dependency is characterized by a marked impairment in an individual’s physiological, psychological, and social functioning due to their uncontrolled usage of mobile phones (Yen et al., 2009). Individuals dependent on their mobile phones may experience negative consequences in both the physical and mental worlds. Research has shown that impaired vision, poor sleep, poor academic performance, lack of physical activity, and an increased risk of physical health concerns are typical somatic symptoms associated with mobile phone dependency (Hong et al., 2019; Dong et al., 2023). Furthermore, studies have shown that an individual’s psychological well-being may suffer due to excessive mobile phone use. These impacts cover a wide range of psychological elements, such as internalizing issues, information overload, sadness, emotional distress, and cognitive failure behaviors (Zhang et al., 2020).

Many academics have recently emphasized that mobile phone dependence is an addictive behavior that does not require drug usage despite the serious hazards to one’s physical and mental health. They have advocated for research into the causes of mobile phone dependence, the elements that support its stability, and its effects on behavior and health. They have also emphasized the critical role that psychological “dependence” or “pathological use” of mobile phones plays in this phenomenon.

According to the fifth National Survey Report on Internet Usage by Minors, which was co-published by the Internet Network Information Centre of China and the Department for Defending the Rights and Interests of Youth of the Central Committee of the Communist Youth League, there will be 193 million underage internet users in China by 2022. Additionally, 91.3% of users rely on mobile phones, which are now the main devices kids and teenagers use to access the Internet, according to the survey. Researchers in psychology, medicine, and other related professions are becoming increasingly concerned about mobile phone dependence as more and more children and teenagers in China use their phones to access the Internet.

The current body of research on mobile phone dependence treatment methods mostly focuses on group psychological intervention (Lan et al., 2018; Wu et al., 2024), solution-focused brief therapy (Xu et al., 2017), mindfulness-based cognitive therapy (Zhang and Zhu, 2014), and inhibition control training (Ding et al., 2018). In recent years, exercise has become a low-consumption, environmentally friendly behavioral health intervention. It serves purposes comparable to cell phones, such as social and leisure activities. At the same time, exercise has been shown to considerably improve people’s psychological self-confidence, physical self-esteem, and ability to alleviate tense and stressful emotions. It has also been shown to improve people’s cognitive capacities, including neurological and somatic capabilities. As a result, academics from various scientific fields increasingly include exercise in treating mobile phone dependence.

Although multiple studies have demonstrated that exercise can significantly increase a child or adolescent’s reliance on mobile phones, there were notable differences in baseline indicators, including scale use, intervention content, intervention intensity, and sample size. Furthermore, there could have been a bias in some of the research. To avoid and treat this condition, assessing the whole intervention effect of exercise prescription on mobile phone dependence in children and adolescents is crucial.

To investigate the relationship between different elements (intervention content, intensity, period, frequency, and length of a single intervention) and the overall effect of exercise on mobile phone dependence in children and adolescents, a meta-analysis was utilized to examine the overall impact of exercise on this topic. To improve the effectiveness of treating mobile phone dependence in kids and teens, another goal was to find an exercise regimen that would have a more improved effect.

## 2 Methods

### 2.1 Search strategy

**Figure 1:**
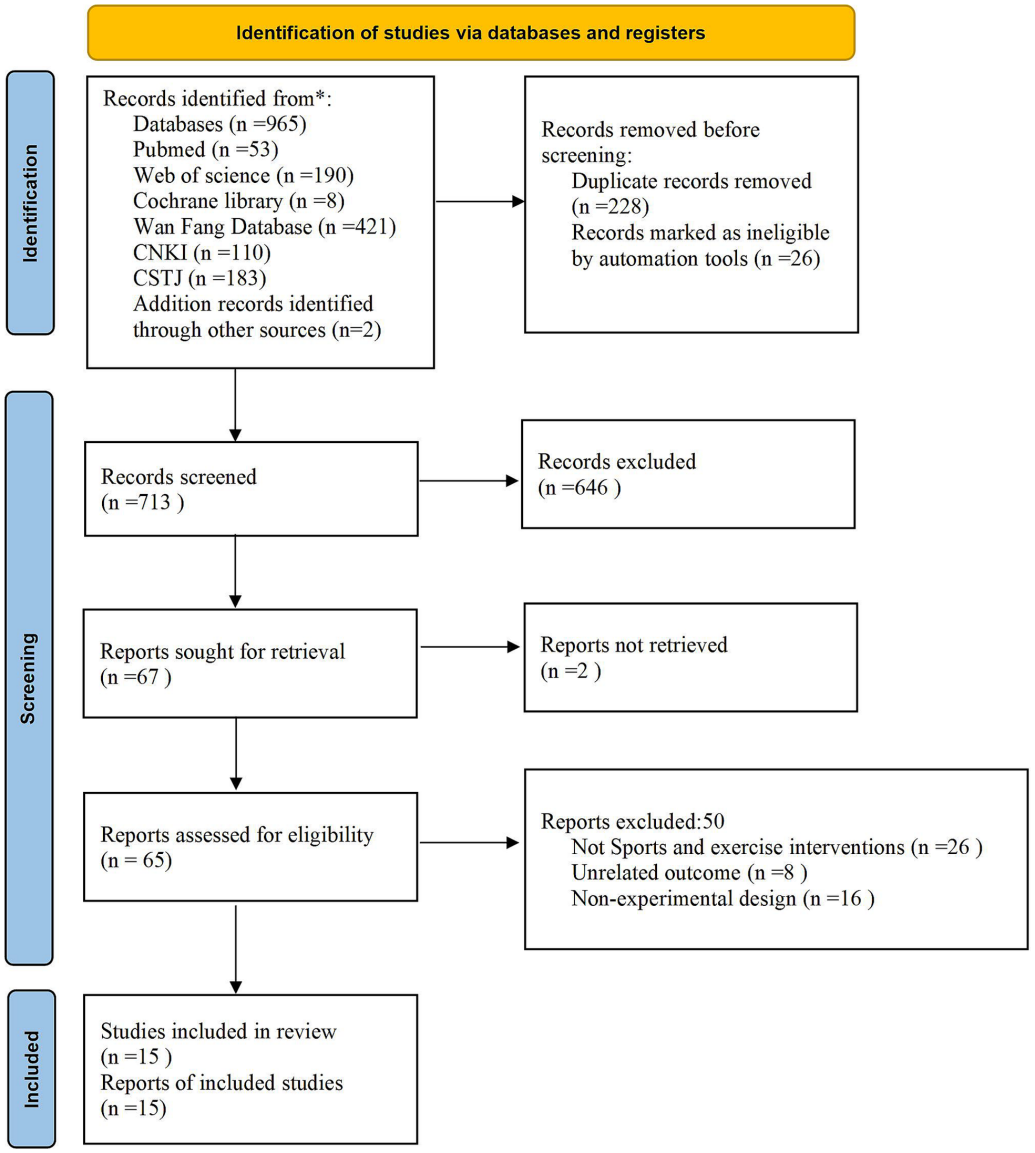
Flow chart of the selection process.

The protocol of this systematic review was registered in PROSPERO (CRD42024543710). A thorough literature search was done using several databases, including the Cochrane Library, PubMed, Web of Science, Wanfang, China Knowledge, and Wipro. The database’s creation date was chosen as the search window, and it ended on December 10, 2023. “Mobile phone addiction,” “smartphone dependence,” “problematic mobile phone use,” “smartphone addiction,” “mobile phone Internet addiction,” “mobile phone game addiction,” “exercise,” “sport*,” “training, physical activity,” “adolescent*,” “sports,” “teens,” “youth,” “child,” “teenager,” “juvenile,” “young,” “minor,” and “student*” were among the English search terms that were used. The Chinese search terms are as follows: “shoujiyilai (手机依赖),” “shoujichengyin (手机成瘾),” “shoujichenmi (手机沉迷),” “shoujiguodushiyong (手机过度使用),” “wentixingshoujishiyong (问题性手机使用),” “zhinengshoujichengyin (智能手机成瘾),” “shoujiwangluochengyin (手机网络成瘾),” “shoujiyouxichengyin (手机游戏成瘾),” “yundongganyu (运动干预),” “yundong (运动),” “tiyuduanlian (体育锻炼),” “shentihuodong (身体活动),” “youyangyundong (有氧运动),” “kangzuxunlian (抗阻训练),” “ertong (儿童),” “qingshaonian (青少年),” and “xuesheng (学生).” A total of 967 documents were obtained after manual searches for references included in the literature and gray literature were carried out. Figure 1 depicts the search procedure.

### 2.2 Literature inclusion and exclusion criteria

#### 2.2.1 Inclusion criteria

The inclusion criteria were strictly based on PICOS criteria (Moher et al., 2009): (1) the subjects were Chinese children and adolescents with mobile phone dependence, ages 6 to 19. (2) The interventions were multifactorial exercise interventions with different content, intensity, duration, frequency and period in the experimental group. (3) The control group did not impose a targeted motor exercise intervention. (4) The outcome indicators were the mean (m), standard deviation (sd) and sample size (n) of the cell phone dependence scores of the experimental and control groups after the intervention. (5) The study design should be a Randomized Controlled Trial (RCT).

#### 2.2.2 Exclusion criteria

The exclusion criteria were as follows: (1) redundant publications; (2) abstract only, without the full text; (3) studies or conference abstracts that are non-direct experimental; (4) no data; (5) use of measuring paradigm endpoint indicators that do not satisfy the requirements of meta-analysis; and (6) not in Chinese or English. A third investigator arbitrated disputes between the two independent investigators who screened the literature.

### 2.3 Data extraction and literature quality assessment

#### 2.3.1 Data extraction

After extracting the essential data from the literature, the two researchers double-checked their findings. To assess interrater reliability, Cohen’s kappa coefficient was calculated to measure the agreement between the two reviewers. Any discrepancies were resolved through discussion with a third researcher. The calculated Cohen’s kappa coefficient was 0.82 (*k* ≥ 0.81: Almost perfect agreement), indicating a strong level of agreement. We then evaluated the quality risk of the literature contained, and a third researcher stepped in if there was a disagreement. Finally, all the researchers agreed to assess the accuracy of the data extraction. First author, year of publication, mobile phone dependency evaluation instrument, sample source, kind of experiment, content of intervention, intensity, period, frequency, single duration of intervention, and outcome evaluation index were the main components of the extraction.

#### 2.3.2 Quality assessment

Two investigators assessed the included studies’ risk of bias using the Risk of Bias Assessment Tool for RCTs from the Cochrane Handbook. Each study’s quality was assessed along the following seven dimensions: (1) generation of randomized sequences; (2) allocation concealment; (3) blinding of the investigators and subjects; (4) blinding of the outcome assessors; (5) completeness of the outcome data; (6) selective reporting of the study results; and (7) other sources of bias. Every level was shown to have a low risk of bias, a high risk of uncertainty, and both. Discussion and agreement with the third investigator were used to resolve discrepancies in the evaluation results.

### 2.4 Data analysis

RevMan 5.4 software was used for several analytical processes, such as data merging, publication bias analysis, heterogeneity testing, forest charting, subgroup analysis, and sensitivity analysis. All of the literature outcome indicators were continuous variables with the same unit of measurement; however, there was a significant disparity in the assessment procedures employed, including scale items and evaluation criteria. Therefore, the standardized mean difference (SMD) and 95% confidence interval (CI) were selected as the effect scale indicators for the combined effect size statistics to evaluate the heterogeneity using the value of *I*^2^ quantitatively. The random-effects model was used to combine the data when *I*^2^ was ≥50%, and additional subgroup analyses were carried out to find the cause of heterogeneity. A fixed-effects analysis was then employed to determine the cause of the heterogeneity. Using a fixed-effects model, subgroup analyses were performed to determine the cause of heterogeneity and vice versa. Results were considered statistically significant when *p* < 0.05.

#### 2.4.1 Sensitivity analysis for excluded studies

To evaluate the potential impact of excluding studies that did not report the specified effect size measures, a sensitivity analysis was conducted. A sensitivity analysis helps determine how robust the results are to certain assumptions or decisions, such as excluding studies that lack the necessary data. By systematically excluding these studies and observing the impact on the overall results, a sensitivity analysis can give a clearer understanding of how the findings might change under different scenarios. Thus, comparisons were made between the pooled effect sizes before and after excluding them. Such analyses guaranteed the synthesized findings were both robust and reliable.

## 3 Results

### 3.1 Literature screening process

After a preliminary search of the databases found 965 similar pieces of literature. Two more pieces were found manually by looking through different sources. Seven hundred and sixteen of the 965 literature were written in Chinese, while 251 were written in English. Seven hundred thirteen relevant pieces of literature remained after 254 duplicate publications were eliminated. After the abstracts and complete texts were first reviewed, 648 studies that were not experimental were removed, yielding 65 experimental research. After reading the entire text again, the studies that did not fit the PICOS inclusion criteria—50 papers that could not be merged for effect sizes—were eliminated. In the end, 15 studies were acquired for the qualitative and meta-analysis (Figure 1).

### 3.2 Basic characteristics of the included studies

Table 1 displays the fundamental attributes of the collected research.

**Table 1:** Basic characteristics of the literature including studies on the effectiveness of exercise as an intervention for mobile phone dependence in children and adolescents.

Inclusion of studies	Type of experiment	Sample sources	Sample size		Gender of the sample	Intervention		Exercise intensity	Intervention period	Intervention frequency	Length of single intervention	Outcome indicator
			Experimental group	Control group		Experimental group	Control subjects					
Zheng et al. (2019)	RCT	Colleges	50	49	Female	Team sports: calisthenics, badminton, rope skipping, basketball, etc.	No intervention with exercise	—	8 weeks	5 times a week	60 min	SADMP
Zhang (2018)	RCT	Colleges	26	26	Male, female	Taiji	Preserving the original way of life	Low intensity	10 weeks	3 times a week	60 min	MPAI
Yang (2022)	RCT	Vocational secondary school	36	36	Male, female	Group K1, K2, K3 aerobics level	Without engaging in any exercise	Medium intensity	12 weeks	3 times a week	30 min	MPAI
Wang (2021)	RCT	Junior high school	21	20	Male, female	Exercises for functional training include a warm-up, strength, speed, coordination, agility, brain activation, aerobic, and relaxation sessions.	Continuing with regular behavioral activities	Medium intensity	30 weeks	2 times a week	40 min	SAS-SV
Wang (2016)	RCT	Colleges	36	37	Male, female	Games involving balls, such volleyball, badminton, table tennis, and basketball	No intervention with exercise	Medium intensity	12 weeks	3 times a week	45 min	SAS-C
Liu (2020)	RCT	Secondary schools	70	70	male, female	Taekwondo, Basketball, and Badminton	Continuing with daily routines	—	10 weeks	For the first 9 weeks, twice a week; by week ten, four times a week	120 min	SAS
Liu et al. (2022)	RCT	Colleges	31	34	Male, female	Basketball	No interventions	Medium intensity	10 weeks	2 times a week	60 min	MPAI
Li (2020)	RCT	Colleges	16	16	male, female	Aerobic exercise on the elliptical, power cycle, treadmill, and badminton	No exercise interventions	Medium intensity	12 weeks	3 times a week	90 min	MPATS
Xie (2019)	RCT	Colleges	162	152	Male, female	Eight pieces of brocade	Received health education	Low intensity	8 weeks	10 times a week	20-30 min	MPAI
Ge et al. (2015)	RCT	Colleges	18	18	Male, female	Volleyball	Continuing with daily routines	—	18 weeks	3 times a week	120 min	MPAI
Du (2021)	RCT	Middle school	20	15	Male, female	Soccer ball	Continuing with daily routines	From medium to high intensity	10 weeks	3 times a week	40 min	MPDM
Zhu (2017)	RCT	Colleges	30	30	Male, female	Twenty-four forms of Taijiquan sets of movements, track and field (fast running), large ball games, health exercises, and yoga are the main exercises to be combined, with no less than two of them in each exercise.	Continuing with daily routines	Medium intensity	8 weeks	3 times a week	60 min	MPATS
Zhang (2023)	RCT	Colleges	30	30	Male, female	Aerobic exercise includes running, basketball, badminton, table tennis, tennis, jumping rope, dancing, etc.	No intervention	From medium to high intensity	8 weeks	3 times a week	60 min	SAS-SV
Jia et al. (2021)	RCT	Colleges	20	20	Male, female	Badminton	Maintaining the original way of life	Medium intensity	10 weeks	2 times a week	60 min	MPAI
Xiao et al. (2021)	RCT	Colleges	31	34	Male, female	Eight pieces of brocade	No exercise intervention	Medium intensity	12 weeks	3 times a week	90 min	MPAI

MPAI is the Mobile Phone Addiction Index Scale; SAS-SV is the Smartphone addiction Scale-Short Version; MPATS is the Mobile Phone Addiction Tendency Scale for College Students; SADMP is the Self-Assessment of Dependence on Mobile Phone for Teenagers questionnaire; SAS-C is the Smartphone Addiction Scale for College Students; MPDM is the Self-Administered Mobile Phone Dependence Measurement Scale for Junior High School Students.

### 3.3 Risk assessment of literature quality

**Figure 2:**
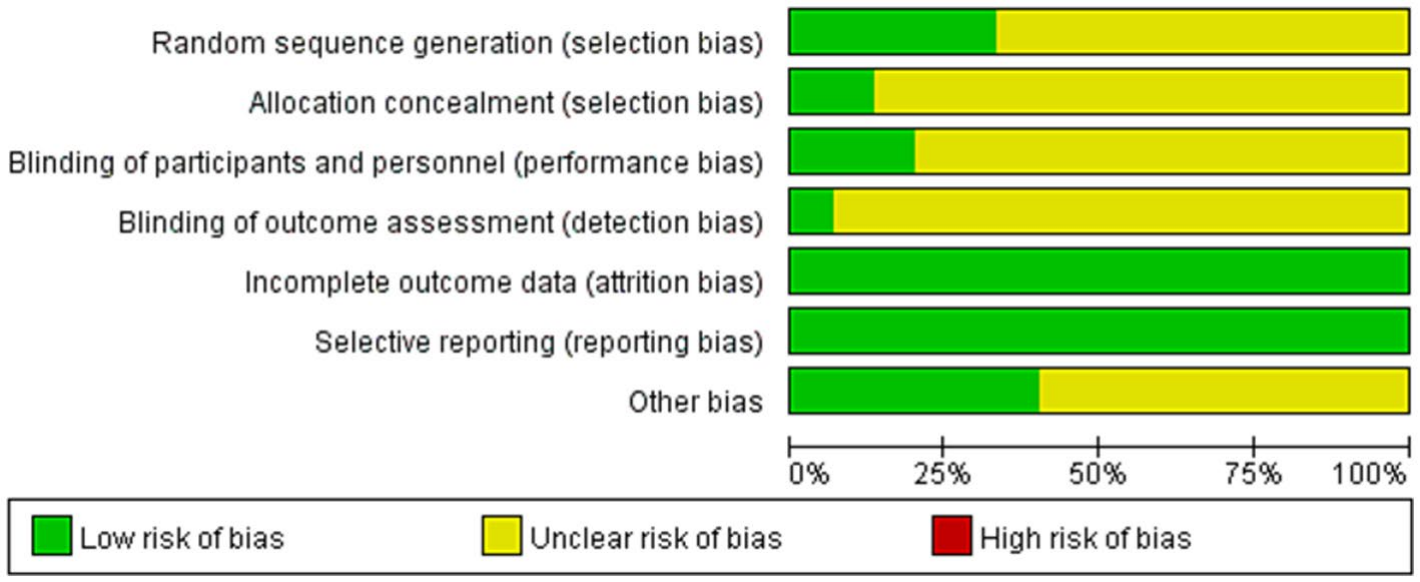
Bias risk of the included studies.

Figure 2 shows a chance of bias in the included research. There were a total of 15 studies; 5 of these included detailed the randomized grouping method, and 2 detailed the allocation concealment scheme. Only 3 of the included studies mentioned the blinding method, while the remaining studies provided an ambiguous explanation. All 15 included studies had complete outcome data.

### 3.4 Meta-analysis of sport exercise intervention for mobile phone dependency in adolescents

#### 3.4.1 Meta-analysis of effect sizes combined results

**Figure 3:**
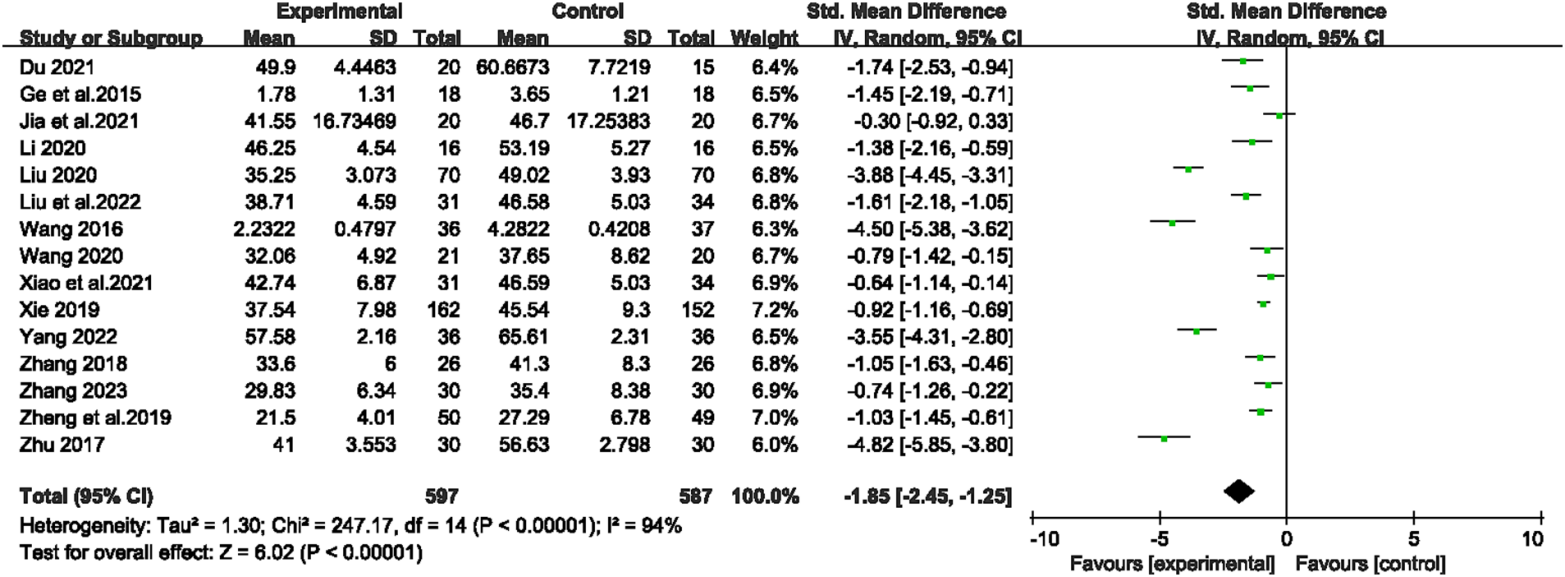
Forest plot of the effect of exercise interventions on mobile phone dependence in children and adolescents.

Using a randomized controlled trial research approach, 15 studies looked at the efficacy of exercise as a holistic solution for children’s and teenagers’ dependence on mobile phones. The research was conducted using the random effects model, and as shown in Figure 3’s results, *I*^2^ = 94%. The total effect size SMD = −1.85, 95% CI (−2.45, −1.25), *p* < 0.000001, on the other hand, demonstrated that the sports and exercise intervention could lessen children’s and teenagers’ dependence on mobile phones.

#### 3.4.2 Analysis of bias

**Figure 4:**
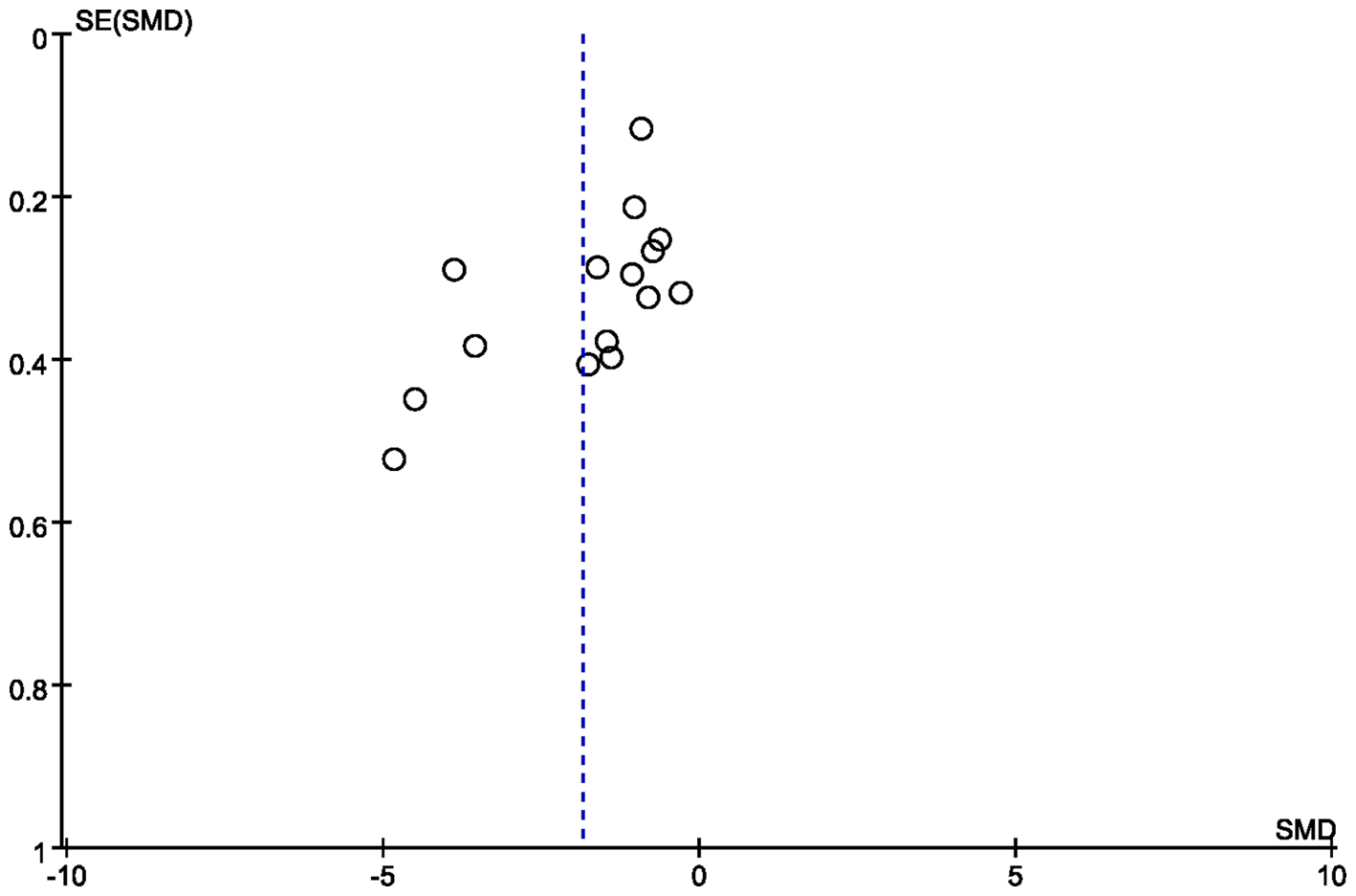
Funnel plots of publication bias.

The study results showed that the funnel plot was symmetrically distributed between left and right, so there was no significant publication bias in this study (Figure 4).

### 3.5 Subgroup analysis of moderator variables in exercise intervention programs

The six components of the exercise program—intervention content, intensity, period, frequency, and duration of one exercise session—were tested in this study using subgroups (Table 1).

#### 3.5.1 Intervention content

There was significant heterogeneity between the effect sizes of the two groups (*I*^2^ = 94%, *p* < 0.05), with a total sample size of 1,184 cases. This suggests that the intervention’s content was the main factor influencing the effectiveness of exercise in treating mobile phone dependence in children and adolescents. The effect size was the largest and statistically significant in the aerobic exercise group [SMD = −1.72 (−2.33, −1.12), *P* < 0.00001]. However, the combined exercise group did not yield a statistically significant result [SMD = −2.78 (−6.74, 1.17), *p* > 0.05]. It can be seen that aerobic exercise has a significant impact on reducing cell phone dependence in children and adolescents.

#### 3.5.2 Intensity of intervention

Three studies did not account for intervention intensity status in detail, with a total sample size of 909 cases included. The SMD [95% CI] of the intervention effect sizes for mobile phone dependence in children and adolescents were −0.97 [−1.18, −0.76] for low intensity, −2.28 [−3.51, −1.04] for high intensity, and −1.19 [−2.16, −0.22] for moderate to high intensity, all of which were statistically significant (*p* < 0.05). The greatest intervention effect was achieved with the moderate intensity of the exercise intervention.

#### 3.5.3 Intervention period

A total sample size of 1,184 cases was included in this variable group, with high heterogeneity among the five subgroup effect sizes (*I*^2^ = 94%, *p* < 0.05), indicating that the intervention period influences the intervention effect of exercise on mobile phone dependence in children and adolescents. The SMD [95% CI] of the intervention effect sizes on mobile phone dependence in child adolescents were −1.73 [−2.69, −0.76] (*p* < 0.05) in the 8-week group, −1.72 [−2.96, −0.48] (*p* < 0.05) in the 10-week group, and −2.50 [−4.31, −0.69] (*p* < 0.05) in the 12-week group, respectively. 18-week and 30-week group there was only 1 group in each, so no between-group comparison could be made. It can be seen that the 12-week group achieved the greatest intervention effect in improving mobile phone dependence in children and adolescents.

#### 3.5.4 Intervention frequency

One study did not provide sufficient detail regarding the frequency of intervention, and a total sample size of 1,044 cases was included, with a high degree of heterogeneity in the effect sizes of the subgroups (*I*^2^ = 92%, *p* < 0.00001). This suggests that the frequency of intervention is an important factor influencing the effectiveness of exercise and movement in intervening in mobile phone dependence in children and adolescents. The SMD [95% CI] of the effect sizes of mobile phone dependence in children and adolescents were −0.91 [−1.68, −0.13] (*p* < 0.05) in the 2-times/week group and −2.17 [−3.11, −1.23] (*p* < 0.05) in the 3-times/week group, respectively. However, there was only one group in the 5-times/week group versus the >5-times/week group, and therefore no group comparisons could be made. The results demonstrate that the three times a week intervention group exhibited the most favorable outcomes.

#### 3.5.5 Duration of one exercise session

The exercise intervention for mobile phone dependence in children and adolescents were conducted in four main groups, with each group lasting between 20 and 90 min. The first group lasted 20 to 30 min, the second 40 to 45 min, the third 60 min and the fourth and longest group lasted at least 90 min. This variable group included 1,184 cases in total. The effect sizes of the four groups showed significant heterogeneity (*I*^2^ = 94%, *p* < 0.00001), suggesting that the duration of a single exercise session was an important factor influencing the effectiveness of the intervention on mobile phone dependence. To explore the potential sources of this variability, a meta-regression analysis was conducted, taking into account variables such as age, gender, and exercise duration. The results indicated a significant correlation between the duration of the exercise session and the size of the intervention’s effect (*p* < 0.05). In contrast, neither age nor gender had a significant influence on the effect size, suggesting that these factors did not substantially contribute to the variability observed. For children and adolescents, the SMD [95% CI] of the intervention effect values were −1.81 [−4.79, 0.36] for the 20–30 min group, −2.32 [−4.43, −0.22] for the 40–45 min group (*p* < 0.05), −1.51 [−2.34, −0.67] for the 60 min group (*p* < 0.05), and −1.84 [−3.41, −0.27] (*p* < 0.05). The 40 ~ 45 min group had the greatest intervention in lowering kids’ and teens’ dependence on cell phones. Furthermore, a sensitivity analysis was conducted to evaluate the effect of individual studies on the overall pooled effect size. The analysis found that no single study had an undue influence on the final outcome, indicating that the conclusions drawn from this analysis are reliable, regardless of whether individual studies are included or excluded (Table 2).

**Table 2:** Results of subgroup analysis of the effects of moderating variables in exercise prescriptions to intervene in mobile phone dependence in children and adolescents.

Moderator variable	Heterogeneity		Subgroup	Number of literature	Sample size	SMD [95%CI]	Two-tailed test	
	*P*	*I*^2^ (%)					*Z*	*P*
Intervention content	<0.001	94	Aerobics	13	1,083	−1.72 [−2.33, −1.12]	5.47	<0.001
			Combinatorial exercise	2	101	−2.78 [−6.74, 1.17]	1.38	0.17
Intensity of intervention	<0.001	93	Low intensity	3	398	−0.97 [−1.18, −0.76]	9.13	<0.001
			Medium intensity	7	416	−2.28 [−3.51, −1.04]	3.61	0.0003
			Medium to high intensity	2	95	−1.19 [−2.16, −0.22]	2.4	0.02
Intervention period	<0.001	94	8 weeks	4	533	−1.73 [−2.69, −0.76]	3.5	0.0005
			10 weeks	5	332	−1.72 [−2.96, −0.48]	2.72	0.007
			12 weeks	4	242	−2.50 [−4.31, −0.69]	2.7	0.007
			18 weeks	1	36	−1.45 [−2.19, −0.71]	3.82	0.0001
			30 weeks	1	41	−0.79 [−1.42, −0.15]	2.42	0.02
Intervention frequency	<0.001	92	2 times/week	3	146	−0.91 [−1.68, −0.13]	2.3	0.02
			3 times/week	9	485	−2.17 [−3.11, −1.23]	4.51	<0.001
			5 times/week	1	99	−1.03 [−1.45, −0.61]	4.82	<0.001
			>5 times/week	1	314	−0.92 [−1.16, −0.69]	7.77	<0.001
Duration of one exercise session	<0.001	94	20 ~ 30 min	2	386	−2.21 [−4.79, 0.36]	1.68	0.09
			40 ~ 45 min	3	149	−2.32 [−4.43, −0.22]	2.16	0.03
			60 min	6	376	−1.51 [−2.34, −0.67]	3.55	0.0004
			≥90 min	4	273	−1.84 [−3.41, −0.27]	2.29	0.02

### 3.6 Sensitivity analysis

Sensitivity analysis is a method used to evaluate the stability and reliability of the results of a meta-analysis or systematic review. In this study, sensitivity analysis was performed on the 15 articles included in the review, primarily through the systematic exclusion of individual studies, modifications to the analytical model, and re-examination of the effect size calculations. The results demonstrated a high degree of stability, indicating that the findings of this meta-analysis are reliable (SMD = −1.85, 95% CI: [−2.45, −1.25]). Furthermore, to address potential publication bias, a subsequent sensitivity analysis was conducted after excluding studies that lacked specified effect size measures. The findings revealed that this exclusion did not significantly alter the overall conclusions of the study, further reinforcing the reliability of the results.

## 4 Discussion

### 4.1 Overall effect analysis of exercise prescription intervention on mobile phone dependence in children and adolescents

The results of the meta-analysis indicated that exercise had a significant impact on reducing mobile phone dependence in children and adolescents. Previous studies have demonstrated a strong correlation between exercise and mobile phone dependence, a finding that aligns with the results of the present study (Wei, 2023; Zhang et al., 2023; Han et al., 2018). Meta-analysis has consistently shown that exercise interventions can significantly improve mobile phone dependence in children and adolescents.

The effectiveness of exercise interventions in reducing mobile phone dependence can be understood through both neurobiological and psychological mechanisms. From the neurobiological view, exercise regulates neurotransmitter systems, dopamine especially. Regular physical activity modulates dopaminergic pathways, it does so by increasing sensitivity to receptors as well as dopamine release in the key brain regions related to reward, impulse, and executive functions (Ma et al., 2022). This dual modulation can eventually reverse internet and mobile phone dependence behavior and improve neural plasticity and cognitive control. In addition, exercise-induced upregulation of brain-derived neurotrophic factor (BDNF) might further increase cognitive flexibility and diminish compulsive behavior related to excessive smartphone use. On a psychological level, exercise serves as an effective behavioral intervention by alleviating stress, reducing anxiety, and enhancing mood through endorphin release and autonomic nervous system regulation. Some research works can generally support the claim that indulging in structured physical activities gives a health-based alternative to screen-based behaviors since it keenly fosters better self-regulation abilities and social interactions while minimizing the need to use mobile devices for emotional coping (Xu, 2019). The present research was in line with these basic findings since the inclusion of exercise interventions significantly reduced mobile dependence likely to accrue with the capacity to bring about both physiological and psychological well-being.

### 4.2 Analysis of the effect of moderating variables of exercise intervention prescription on mobile phone dependence in children and adolescents

#### 4.2.1 Content of intervention

The results of the study demonstrated that aerobic exercise had a notable impact on reducing mobile phone dependence in children and adolescents. Conversely, combined exercise exhibited a non-significant intervention effect on mobile phone dependence in this study. Based on the observed changes in physical function in children and adolescents and the controllable perspective of exercise risk, aerobic exercises such as small and large ball games, group aerobics, running, yoga, rope skipping, Tai Chi, and Eight pieces of brocade represent the most commonly used types of exercise content for children and adolescents. Prior research has demonstrated that aerobic exercise can effectively mitigate the psychological craving and addiction level of individuals with mobile phone dependence (Zhao et al., 2021). The results of this study are supported by the findings that a variety of aerobic exercise interventions are used to meet the different needs of students, stimulate their interest in exercise and internal drive, and promote their basic physical mobility and adaptive function in the natural environment. Furthermore, aerobic exercise has been shown to regulate behavior and improve interpersonal skills, which can lead to a reduction in the level of mobile phone dependence (Zhu, 2017).

The effectiveness of aerobic exercise in reducing mobile phone dependence can also be explained through dual-process theories in addiction research. The theories suggest that addictive behaviors result from imbalances between impulsive, reward-driven processes and reflective, goal-directed self-control mechanisms. In adolescents, there is a high dominance of impulsive processes such that compulsive phone uses take over while the reflective process fights to inhibit those compulsions. Aerobic exercise helps restore this balance by strengthening top-down inhibitory control, particularly in the prefrontal cortex, which regulates impulsive behaviors and enhances self-control. Furthermore, aerobic exercise decreases automatic approach biases to reward stimuli—unconscious tendencies to approach positive stimuli—thus weakening the reflexive impulsive mobile phone checking urge. Cognitive and neurobiological mechanisms combine, hence aerobic exercise may well be an effective and scalable intervention for most adolescents in mitigating excessive use of the mobile phone.

#### 4.2.2 Intensity of intervention

The findings of this study indicate that a medium intensity intervention was the most effective in reducing mobile phone dependence among children and adolescents. The moderate to high intensity group exhibited the greatest intervention effect, while the low intensity group demonstrated the lowest level of intervention effect. As evidenced by the findings of previous experiments, acute aerobic exercise at moderate intensity has been demonstrated to facilitate inhibition and significantly enhance cognitive processing efficiency (Wang and Zhou, 2014; McMorris and Hale, 2012). The results of the analysis indicated that this phenomenon can be observed from a neurophysiological perspective. Long-term participation in moderate-intensity exercise has been demonstrated to effectively increase the level of neuronal activation and physiological arousal, thereby improving the efficiency of resource allocation. Furthermore, it has been shown to significantly promote inhibitory capacity, which may contribute to a reduction in the degree of mobile phone dependence in children and adolescents (Wu et al., 2023). Furthermore, it has been demonstrated that sports exercise, particularly moderate-intensity exercise, can effectively prevent and treat internet addiction (Ke and Zhou, 2015; Weinstock et al., 2017). Meanwhile, previous quantitative studies on students’ physical exercise and mobile phone dependence have indicated that moderate-intensity exercise exerts the optimal positive effect on college students’ mobile phone dependence, in comparison with low and high exercise (Yang et al., 2020). The findings of this study align with those of previous research, providing further evidence that moderate-intensity exercise is an effective intervention for reducing mobile phone dependence.

#### 4.2.3 Single intervention duration, frequency, and period of intervention

The maximum intervention effect was observed in the group that engaged in a single exercise session lasting 40–45 min. The optimal exercise frequency was three times per week, aligning with the World Health Organization’s recommendations, with a minimum frequency of two sessions per week. A 12-week intervention cycle demonstrated the most significant effect on reducing mobile phone dependence, followed by an 8-week cycle. However, the effect size of the exercise intervention on mobile phone dependence progressively diminished with longer intervention durations. This suggests a diminishing return effect, whereby prolonged intervention cycles may not yield additional benefits. In line with these findings, previous research by Jaworska et al. (2019) on 12 medication-naïve adolescents involved a 12-week aerobic exercise program consisting of three sessions per week, each lasting 45 min, including a 5-min warm-up, 30 min of aerobic exercise, and a 10-min cool-down/stretching session. This intervention led to significant reductions in depressive symptoms, which may have contributed to alleviating mobile phone dependence (Li et al., 2023). However, while the 12-week cycle demonstrated the strongest effects, the long-term sustainability of these benefits remains unclear. To better inform future interventions, it is crucial to explore whether these improvements are maintained over extended periods, and further longitudinal research is needed to assess the enduring impact of exercise on mobile phone dependence.

#### 4.2.4 The risk role of parenting styles and behavioral control in mobile phone dependence

Despite the efficacy of exercise interventions, a deeper understanding of the risk factors for mobile phone dependence, particularly among children and adolescents, is necessary. The literature suggests that mobile phone dependence results from numerous psychosocial factors, mainly parenting styles and their levels of behavioral control. For instance, a meta-analysis on adolescent problematic internet use (PIU) reported weak negative correlations between PIU and parenting practices such as warmth, control, and authoritative parenting (Lukavská et al., 2022). Notably, younger adolescents’ PIU was not significantly associated with restrictive media mediation, whereas older adolescents’ PIU was positively correlated, indicating that overprotective parenting might unintentionally perpetuate misuse of technology. Similarly, a cross-sectional study involving a South Korean cohort reported that parents’ control of smartphone access contributed to greater smartphone addiction, especially in children aged 10–12 years (Lee and Ogbolu, 2018). Excessive parental control that exceeds technological boundaries could exacerbate smartphone addiction among children, as reported by this study. Moreover, another study conducted on adolescents’ online gaming behavior found that parental mediation and interpersonal interactions play significant roles in the development of problematic gaming behavior. More parental supervision correlated with a lesser tendency for adolescents to use video games to escape from negative feelings. In contrast, overly controlling mediation was found to be correlated with lower social skills in adolescents, including higher levels of impulsive behavior, social anxiety, and stress. These lower social skills increased the chances of using escape through gaming (Commodari et al., 2024). From the results, the parents’ approach to management and control of mobile phone usage could use more open and communicative models that may be more effective in combating mobile phone dependence. So, most likely, more research is needed on how different parenting styles and exercise strategies can be combined for more effective prevention of mobile phone dependence.

#### 4.2.5 Limitations

This study examined literature based on the included studies’ Cochrane classification biases using the Cochrane Handbook’s Risk of Bias Assessment Tool for RCTs. Even after two different researchers independently scored multiple iterations, some bias could still arise from subjectivity. Further research should apply more tools for evaluating the literature’s quality to reduce subjective bias and increase reliability.

As a meta-analysis research paper, this study is based on existing completed studies and, therefore, will inevitably be limited and influenced by some non-controllable conditions. The quantity and quality of the included literature is the primary basis for determining the quality of the Meta-analysis and the main basis for the objectivity of the study’s conclusions. The quality of the included studies in this paper is mixed, especially in the methodological part of the study design, ambiguities about randomization, allocation concealment and blinding implementation may affect the reliability of the conclusions, but we used the methods of sensitivity analysis and bias analysis to increase the stability and reliability of the conclusions.

Additionally, this study did not differentiate between varying levels of mobile phone dependence (mild, moderate, and severe), limiting the ability to assess whether exercise interventions have differential effects across severity levels. Future research should consider stratified analyses to examine how exercise prescriptions impact individuals with different levels of dependence. Moreover, there is a need for large-scale, multi-center, standardized, and methodologically rigorous studies to strengthen the evidence base for exercise interventions in addressing mobile phone dependence among children and adolescents.

Furthermore, while previous meta-analyses on behavioral addictions examined different intervention strategies, only a few investigated, in a thorough manner, the role of exercise in the reduction of mobile phone dependence. The comparison of the current findings with earlier meta-analyses on internet addiction, gaming disorder, and other behavioral addictions could provide meaningful information about the distinct and overlapping mechanisms that underlie these disorders. Future studies should explore how exercise interventions compare with other behavioral therapies in the management of digital addiction, yielding a holistic perspective into the effective strategies of treatment.

## 5 Conclusion

(1) Exercise can effectively promote the improvement of mobile phone dependence among children and adolescents; (2) the content, intensity, period, frequency, and single duration of exercise all affect the intervention effect of exercise on mobile phone dependence among children and adolescents to different degrees, among which the moderate-intensity aerobic exercise that is performed three times a week, with each exercise session lasting for 40–45 min, and lasting for a total of about 12 weeks, is more likely to obtain the desired intervention effect of improving mobile phone dependence among children and adolescents.

Future research can try to start from the following aspects: (1) Improve the comprehensive intervention effect. Explore the exercise dose threshold that promotes the improvement of mobile phone dependence and maintains the intervention effect of mobile phone dependence, so as to improve the comprehensive intervention effect of exercise on children and adolescents; focus on the two-way virtuous cycle of improving mobile phone dependence and promoting physical and mental health, so as to improve the comprehensive health effect of exercise on children and adolescents. (2) Improving the adaptability of exercise interventions. Starting from the intrinsic mechanism and mediating effect of exercise intervention for mobile phone dependence, and targeting children and adolescents with different physical and mental health conditions and at different age levels, we will systematically develop a refined daily practice exercise intervention program and guidelines suitable for the characteristics of mobile phone dependence in different groups of children and adolescents. (3) Improve the design of intervention experiments. The elements affecting mobile phone dependence in children and adolescents are diversified, and from a methodological perspective, it is necessary to standardize the experimental and measurement protocols for mobile phone dependence in children and adolescents, for example, by determining the specific parameters of the exercise intensity level, the specific allocation of intervention time, the exercise habits of the subjects, their family situation, the data from the male and female tests, and the combination of subjective mobile phone use and objective parameter tests.

## Data Availability

The original contributions presented in the study are included in the article/supplementary material, further inquiries can be directed to the corresponding author.
